# Genome engineering using CRISPR/Cas: getting more versatile and more precise at the same time

**DOI:** 10.1186/s13059-016-0922-3

**Published:** 2016-03-17

**Authors:** Holger Puchta

**Affiliations:** Botanical Institute II, Karlsruhe Institute of Technology, Hertzstrasse 16, Karlsruhe, 76187 Germany

## Abstract

A report on the second meeting on ‘Genome Engineering and Synthetic Biology: Tools and Technologies’, held 28–29 January 2016 in Ghent, Belgium.

## Introduction

Over 400 scientists met at the end of January 2016 in the beautiful town of Ghent, Belgium. The well-organized meeting was the second in a series that aims to focus on genome engineering and synthetic biology. Synthetic biology has been a hot topic for a number of years. The recent development of tools for engineering utilizing clustered regularly-interspaced short palindromic repeats (CRISPR) coupled with the CRISPR-associated (Cas) nuclease (so-called CRISPR/Cas technology), and their growing applications, are currently revolutionizing biology. Not surprisingly, during the meeting CRISPR/Cas outshined all other topics. This led to some speakers excusing themselves for not talking about CRISPR/Cas technology. Thus, this Meeting Report will mainly highlight recent progress in genome engineering.

## The CRISPR/Cas revolution is gaining speed

Meeting hundreds of enthusiastic scientists working with CRISPR/Cas technology, it was hard to believe that the conference was taking place only three years after the first reports had been published that revealed that the CRISPR/Cas system can be used as an efficient tool for genome engineering in higher eukaryotes.

It was a sound decision by the organizers to start the meeting with a plenary talk by Jin-Soo Kim from the Seoul National University, Korea. His talk nicely introduced some of the currently most discussed aspects of the CRISPR/Cas technology, such as detection and elimination of off-side effects, as well as broadening its application. He presented a new method, called ‘multiplex Digenome-seq’, to profile genome-wide off-target effect specificities of multiple CRISPR-Cas9 nucleases simultaneously. The technique is based on the digestion of cell-free human genomic DNA, followed by whole-genome sequencing. Another option for reducing off-target effects is to tightly control enzyme activity. Instead of transforming DNA encoding the Cas9 nuclease and the single guide RNA (sgRNA), preassembled complexes of purified Cas9 protein and sgRNA had been transfected successfully by the Korean scientists into cells. Thus, continuous expression of the enzyme, which would enhance off-target effects over time, can be avoided. Notably, this approach is not only attractive for mammalian cells but additionally for crop plants—such plants never come into contact with transgenic DNA, but contain small insertions or deletions that are indistinguishable from naturally occurring mutations. Hopefully, these plants will not be regarded as genetically modified organisms (GMOs) around the globe.

Not only in biotechnology, but also in medicine, new avenues can be taken by applying CRISPR/Cas: hemophilia A is an X-linked genetic disorder caused by mutations in the gene encoding the blood coagulation factor VIII, which are often chromosomal inversions. Kim’s group are now (or were) able to revert these chromosomal inversions in induced pluripotent stem cells (iPSCs) derived from patients. Thus, in the long-run, CRISPR/Cas-based therapeutic applications will become an option to fight this disease.

## Understanding the structure and improving the precision of Cas9

A way to improve the specificity of Cas9 is to redesign the enzyme. To do so, one has to understand the molecular structure in detail. In his presentation, Martin Jinek, a pioneer in the CRISPR/Cas field, from the University Zürich, Switzerland, gave an overview of the latest results on the crystal structure of *Streptococcus pyogenes* Cas9 in complex with an sgRNA and a target DNA containing a canonical protospacer adjacent motif (PAM). The structure revealed that the PAM motif resides in a base-paired DNA duplex, indicating a PAM-dependent target DNA melting and RNA–DNA hybrid formation. Using this structure, rational engineering of Cas9 enzymes with improved specificity or novel PAM sequences is possible.

Benjamin Kleinstiver of Keith Joung’s group from the Harvard Medical School in Boston, USA, showed exactly that—he developed SpCas9-HF1, a high-fidelity variant harboring alterations designed to reduce non-specific DNA contacts. SpCas9-HF1 retains on-target activities comparable to those of wild-type SpCas9. Notably, use of SpCas9-HF1 rendered nearly all off-target events undetectable. One does not have to be a prophet to predict that SpCas9-HF1 will outcompete wild-type Cas9 enzymes, especially for therapeutic applications. Kleinstiver also used molecular evolution to modify the PAM of *Staphylococcus aureus* Cas9 (SaCas9). A variant was identified that showed robust genome editing activities at endogenous human target sites with modified PAMs, thereby increasing the SaCas9 targeting range by twofold to fourfold.

## CRISPR/Cas-mediated genome engineering is becoming more versatile

Randal Platt from Feng Zhang’s group of the Board Institute in Cambridge, USA, reported on how CRISPR/Cas is revolutionizing mouse genetics. The group has established Cas9-expressing mice. By using adeno-associated virus-, lentivirus- or particle-mediated delivery of guide RNA, induction of mutations can be achieved in neurons, immune cells and endothelial cells. Using these mice, simultaneous mutations in *KRAS*, *p53* and *LKB1* (encoding serine/threonine-protein kinase STK11)—the three most prominent genes mutated in lung adenocarcinoma—were obtained. The respective animals developed macroscopic tumors of adenocarcinoma. This highlights that these Cas9 mice represent a tremendous improvement in both time and efficiency in obtaining animal models for carcinogenesis, in comparison with the classical knockout technology.

How far human disease modeling can be taken was demonstrated by Weizhi Ji from Kunming Biomed International, Kunming, China. His group is using synthetic nucleases to induce mutations in rhesus and cynomolgus monkeys. He has been able to reproduce different human disease syndromes by microinjection of plasmids or RNAs coding for the respective synthetic nucleases. Thus, monkeys can be used as an important model species for studying human diseases and developing therapeutic strategies. However, this kind of research brings us closer to the question as to what point, owing to ethical considerations, the application of the technology should be limited.

## Epigenome-editing is breaking new ground

Although applications for genome engineering by CRISPR/Cas were the central focus of the meeting, other applications of the technology are also extremely promising. Charles Gersbach from the Duke University, Durham, USA, summarized the work of his group using transcription activator-like effectors (TALEs) and CRISPR/Cas for epigenome editing. A CRISPR-Cas9-based acetyltransferase, consisting of the nuclease-null dCas9 protein fused to the catalytic core of the human acetyltransferase p300, has been constructed by the group. The idea was that the fusion protein catalyzes acetylation of histone H3 lysine 27 at its target sites, leading to robust transcriptional activation of target genes. Indeed, Gersbach’s group could show that activation of the gene(s) was highly specific. These results demonstrate that targeted acetylation of histones can induce transactivation and is a robust tool for manipulating gene regulation. Furthermore, they showed that fusions of nuclease-inactive dCas9 to the Krüppel-associated box (KRAB) repressor (dCas9-KRAB) can silence target gene expression. dCas9-KRAB was targeted to the HS2 enhancer, a distal regulatory element that orchestrates the expression of multiple globin genes. A highly specific induction of H3K9 trimethylation (H3K9me3) at the enhancer, and decreased chromatin accessibility of both the enhancer and its promoter targets, were detected. These targeted epigenetic modifications of HS2 correlated with the repression of multiple globin genes. Owing to the ability to multiplex targets, the CRISPR/Cas system has the potential to redefine complex gene expression patterns, especially if different dCas9 orthologs are simultaneously applied in a single cell.

## Synthetic biology—getting faster, becoming smaller and pepping up sex lives

Exciting developments were not only reported in relation to the application of the CRISPR/Cas technology, but also for other fields of synthetic biology. Emily LeProust, co-founder of Twist Bioscience, San Francisco, USA, reported on a new strategy for making DNA synthesis much faster and cheaper than before. The company has developed a semiconductor-based synthetic DNA manufacturing process, featuring a 10,000-well silicon platform. By synthesizing DNA on silicon instead of traditional 96-well plastic plates, the platform enables cost-effective, rapid, high-quality and high-throughput synthetic gene production.

The availability of synthetic DNA at a much lower cost will be a major advance for different fields of synthetic biology, but especially for the set-up of synthetic genomes. The major breakthrough in this area was synthesis and assembly of the one-megabase-pair *Mycoplasma mycoides* genome and its transplantation into a *M. capricolum* recipient cell by Craig Venter and colleagues in 2010. Daniel Gibson, inventor of the well-known ‘Gibson Assembly’ technique, from the J. Craig Venter Institute in La Jolla, USA, made a presentation about making this synthetic genome even smaller. Through identification of individually deletable regions, by transposon mutagenesis, and progressively clustering deleted genomic segments, smaller mycoplasmal genomes were obtained. The project is still ongoing, but, as a significant part of the original synthetic genome could already be eliminated, the analysis is obviously coming closer to its aim of obtaining the smallest possible genome for a living organism.

Besides synthesizing artificial genomes, synthetic biology is developing synthetic entities as novel kinds of detectors and temporally and spatially tightly controllable inducers. One can combine these modalities to develop novel signal-integration circuits. Martin Fusssenegger from the ETH Zürich, Switzerland, talked about the control of physiological processes by optogenetic devices that might provide novel treatment opportunities for therapies. His group has developed an erectile optogenetic stimulator (EROS), a synthetic designed guanylate cyclase, producing a blue-light-inducible rush of the second-messenger cyclic guanosine monophosphate (cGMP) in mammalian cells, which enables blue-light-dependent penile erection after illumination in male rats.

## Concluding remarks

Without a shadow of doubt, most participants were surprised and excited by the wealth of novel applications of synthetic biology and genome engineering on show and left the meeting with the feeling that there will be much more progress in the near future.

## Abbreviations

Cas: CRISPR associated
CRISPR: Clustered regularly-interspaced short palindromic repeat
EROS: Erectile optogenetic stimulator
GMO: Genetically modified organism
H3K9me3: Histone H3 lysine 9 trimethylation
iPSC: Induced pluripotent stem cell
KRAB: Krüppel-associated box
PAM: Protospacer adjacent motif
sgRNA: Single guide RNA
TALE: Transcription activator-like effector

